# The relationship between mental-health-related stigma among psychiatrists and country indicators across Europe

**DOI:** 10.1192/j.eurpsy.2024.176

**Published:** 2024-08-27

**Authors:** D. Őri, P. Szocsics, T. Molnár, L. Bankovska Motlova, O. Kazakova, S. Mörkl, M. Wallies, M. Abdulhakim, S. Boivin, K. Bruna, C. Cabaços, E. A. Carbone, E. Dashi, G. Grech, S. Greguras, I. Ivanovic, K. Guevara, S. Kakar, K. Kotsis, I. M. I. Klinkby, J. Maslak, S. Matheiken, A. Mirkovic, N. Nechepurenko, A. Panayi, A. T. Pereira, E. Pomarol-Clotet, S. Raaj, P. Rus Prelog, J. Soler-Vidal, R. Strumila, F. Schuster, H. Kisand, A. Reim, G. Ahmadova, M. Vircik, H. Yilmaz Kafali, N. Grinko, Z. Győrffy, S. Rózsa

**Affiliations:** ^1^ Semmelweis University; ^2^Heim Pal National Pediatric Institute, Budapest; ^3^Aladar Petz County Teaching Hospital, Győr, Hungary; ^4^Charles University, Prague, Czech Republic; ^5^Psychiatric Clinic of Minsk City, Minsk, Belarus; ^6^Medical University of Graz, Graz, Austria; ^7^Therapie auf Augenhoehe, Buelach, Switzerland; ^8^Vrije Universiteit Brussel, Brussels, Belgium; ^9^EPSM du Finistère Sud, Quimper, France; ^10^State Psychiatric Hospital Gintermuiza, Jelgava, Latvia; ^11^ Centro Hospitalar e Universitário de Coimbra; ^12^ Coimbra University; ^13^Coimbra Institute for Biomedical Imaging and Translational Research, Coimbra, Portugal; ^14^University Magna Græcia of Catanzaro, Catanzaro, Italy; ^15^University Hospital Center “Mother Theresa”, Tirane, Albania; ^16^Mount Carmel Hospital, Attard, Malta; ^17^University Hospital Centre Zagreb, Zagreb, Croatia; ^18^Clinical Centre of Montenegro, Podgorica, Montenegro; ^19^State Psychiatric Hospital for Treatment of Drug Addiction and Alcoholism, Sofia, Bulgaria; ^20^Erasmus University Medical Center, Rotterdam, Netherlands; ^21^University of Ioannina, Ioannina, Greece; ^22^Capital Region of Denmark, Copenhagen, Denmark; ^23^Institute for Mental Health, Belgrade, Serbia; ^24^Pennine Care NHS Foundation Trust, Oldham, United Kingdom; ^25^University Children’s Hospital, University Medical Centre Ljubljana, Ljubljana, Slovenia; ^26^The Serbsky State Scientific Center for Social and Forensic Psychiatry, Moscow, Russian Federation; ^27^Freelancer, Larnaca, Cyprus; ^28^FIDMAG Germanes Hospitalàries Research Foundation; ^29^Instituto de Salud Carlos III, Barcelona, Spain; ^30^South Meath Mental Health Service, Co.Meath, Ireland; ^31^University Psychiatric Clinic Ljubljana, Ljubljana, Slovenia; ^32^Complex Assistencial Salut Mental, Sant Boi de Llobregat, Spain; ^33^CHU Montpellier; ^34^University of Montpellier, Montpellier, France; ^35^Vilnius University, Vilnius, Lithuania; ^36^Technische Universität München, Munich, Germany; ^37^University of Tartu, Tartu, Estonia; ^38^United City Hospital N15, Baku, Azerbaijan; ^39^Psychiatric Hospital Michalovce, Mihalovce, Slovakia; ^40^Sultanbeyli State Hospital, Istanbul, Türkiye; ^41^Ukrainian Catholic University, Lviv, Ukraine; ^42^Károli Gáspár University of the Reformed Church, Budapest, Hungary

## Abstract

**Introduction:**

Mental health-related stigma occurs not only within the public community but is also an issue among healthcare professionals. The relationship between national culture and provider stigma remains yet to be empirically attested.

**Objectives:**

We performed a cross-sectional multicentre study across 32 European countries to investigate the attitudes of psychiatrists towards patients with mental health problems. We aimed to examine the relationship of attitude with country-specific indicators.

**Methods:**

We measured stigmatizing attitudes using the Opening Minds Stigma Scale for Health Care Providers (OMS-HC) within an online survey among specialists and trainees in general adult, child and adolescent psychiatry. Its total score was correlated with the Human Development Index (HDI), the Democracy Index (DI), the Social Progress Index (SPI), the number of psychiatrists per 100,000 people, and the Hofstede dimensions. Latent class analysis was done to find subgroups of countries according to the stigmatizing attitudes of psychiatrists and the six Hofstede dimensions.

**Results:**

Altogether, n=4245 participants completed the survey. The total score of the OMS-HC significantly correlated with the long-term orientation (r=0.453, p=0.015) and indulgence dimensions (r=-0.629, p<0.0001) and with the HDI (r=-0.503, p=0.005), DI (r=-0.418, p=0.024), SPI (r=-0.348, p=0.040). The latent class analysis separated high- and low-stigma countries. High stigma was associated with high power distance and uncertainty scores.

**Conclusions:**

Findings from this study not only expand knowledge of factors related to stigmatizing attitudes of healthcare professionals, but also enlighten the cultural aspects of the stigma that could contribute to the further development of anti-stigma programs.

**Disclosure of Interest:**

D. Őri Grant / Research support from: Research grant form the Fulbright Association, P. Szocsics: None Declared, T. Molnár: None Declared, L. Bankovska Motlova: None Declared, O. Kazakova: None Declared, S. Mörkl: None Declared, M. Wallies: None Declared, M. Abdulhakim: None Declared, S. Boivin: None Declared, K. Bruna: None Declared, C. Cabaços: None Declared, E. A. Carbone: None Declared, E. Dashi: None Declared, G. Grech: None Declared, S. Greguras: None Declared, I. Ivanovic: None Declared, K. Guevara: None Declared, S. Kakar: None Declared, K. Kotsis: None Declared, I. Klinkby: None Declared, J. Maslak: None Declared, S. Matheiken: None Declared, A. Mirkovic: None Declared, N. Nechepurenko: None Declared, A. Panayi: None Declared, A. Pereira: None Declared, E. Pomarol-Clotet: None Declared, S. Raaj: None Declared, P. Rus Prelog: None Declared, J. Soler-Vidal: None Declared, R. Strumila: None Declared, F. Schuster: None Declared, H. Kisand: None Declared, A. Reim: None Declared, G. Ahmadova: None Declared, M. Vircik: None Declared, H. Yilmaz Kafali: None Declared, N. Grinko: None Declared, Z. Győrffy: None Declared, S. Rózsa: None Declared

